# Prognostic value of initial chest CT findings for clinical outcomes in patients with COVID-19

**DOI:** 10.7150/ijms.48281

**Published:** 2021-01-01

**Authors:** Song Liu, Chen Nie, Qizhong Xu, Hong Xie, Maoren Wang, Chengxin Yu, Xuewen Hou

**Affiliations:** 1 Department of Radiology, The First College of Clinical Medical Science, China Three Gorges University, Yichang Central People's Hospital, Yichang, China.; 2 Department of Radiology, Yichang Second People's Hospital, Yichang, China.; 3 Department of Radiology, Shenzhen Second People's Hospital, Shenzhen, China.; 4 Department of Ophthalmology, University Medical Center, Johannes Gutenberg University Mainz, Mainz, Germany.; 5 Department of Internal Medicine, Charité-Universitätsmedizin Berlin, German Heart Center Berlin, Berlin, Germany.

**Keywords:** COVID-19, SARS-CoV-2, CT, clinical outcomes

## Abstract

**Rationale:** To identify whether the initial chest computed tomography (CT) findings of patients with coronavirus disease 2019 (COVID-19) are helpful for predicting the clinical outcome.

**Methods:** A total of 224 patients with laboratory-confirmed COVID-19 who underwent chest CT examination within the first day of admission were enrolled. CT findings, including the pattern and distribution of opacities, the number of lung lobes involved and the chest CT scores of lung involvement, were assessed. Independent predictors of adverse clinical outcomes were determined by multivariate regression analysis. Adverse outcome were defined as the need for mechanical ventilation or death.

**Results:** Of 224 patients, 74 (33%) had adverse outcomes and 150 (67%) had good outcomes. There were higher frequencies of more than four lung zones involved (73% vs 32%), both central and peripheral distribution (57% vs 42%), consolidation (27% vs 17%), and air bronchogram (24% vs 13%) and higher initial chest CT scores (8.6±3.4 vs 5.4±2.1) (*P* < 0.05 for all) in the patients with poor outcomes. Multivariate analysis demonstrated that more than four lung zones (odds ratio [OR] 3.93; 95% confidence interval [CI]: 1.44 to 12.89), age above 65 (OR 3.65; 95% CI: 1.11 to 10.59), the presence of comorbidity (OR 5.21; 95% CI: 1.64 to 19.22) and dyspnea on admission (OR 3.19; 95% CI: 1.35 to 8.46) were independent predictors of poor outcome.

**Conclusions:** Involvement of more than four lung zones and a higher CT score on the initial chest CT were significantly associated with adverse clinical outcome. Initial chest CT findings may be helpful for predicting clinical outcome in patients with COVID-19.

## Introduction

Coronavirus disease 2019 (COVID-19) has become a global pandemic caused by a novel virus, severe acute respiratory syndrome coronavirus 2 (SARS-CoV-2) 1,2. As of May 4, 2020, this virus has caused more than 3,500,000 confirmed cases and 240,000 deaths worldwide 3. The COVID-19 outbreak applied extreme stress on the health care systems of most countries. Therefore, determination of prognostic factors for clinical outcomes would be important for relieving this stress and reducing the mortality rate.

Chest high-resolution computed tomography (CT) has become increasingly important for establishing the diagnosis of COVID-19 4-6. The reported CT manifestations mainly included ground-glass opacities (GGOs) and consolidation, with predominantly peripheral distribution and multiple lung zones involvement 7,8. Previous studies involving patients with SARS 9 and influenza A (H1N1) 10 have indicated that chest radiographs might be helpful in predicting clinical outcome. However, whether the initial chest CT findings can help clinicians to predict the clinical outcomes in patients with COVID-19 remains unclear.

Thus, this study sought to investigate whether the initial chest CT findings of patients with COVID-19 are helpful for predicting the clinical outcome.

## Methods

### Study design and population

This retrospective study was performed in Yichang Central People's Hospital, Yichang, Hubei province, China. The institutional review board approved this study, and the requirement for informed consent was waived due to its observational nature. Between January 10, 2020, and March 28, 2020, 224 consecutive patients with laboratory-confirmed COVID-19 by reverse transcription-polymerase chain reaction test, with chest CT examinations within the first day of admission were included in the study. The electronic medical records of patients with COVID-19, including clinical symptoms, laboratory data, and clinical outcomes, were extracted and analyzed by research team. Adverse clinical outcomes were defined as the need for mechanical ventilation or death. Good clinical outcomes were defined as no mechanical ventilation or death.

### Chest CT acquisition and image analysis

All chest CT examinations were obtained using a SOMATOM Definition FLASH 16-array scanner (SIEMENS, Germany) within the first day of admission. The scanning range was from the tip of the lung to the bottom of the lung. The specific parameters were as follows: tube voltage (130 kV), tube current (100 mAs), slice thickness (1.5 mm), and interval (1.5 mm). All CT images were assessed by two radiologists (S.L., and C.N., with 8 and 10 years of clinical experience, respectively) who were blinded to the clinical findings, and final decisions were reached by consensus.

The distribution (central, peripheral and mixed) and pattern (GGO, consolidation, and air bronchogram) of lung abnormalities on initial CT were assessed and analyzed. Location and number of lung zones involved were recorded. The definitions of peripheral location, central location, ground glass opacities and consolidation were as previously described 11. The extent of lung abnormalities on initial CT was assessed by a semi-quantitative scoring system 12. Each lung was classified into three lung regions: the upper lung zone (above the carina), the lower lung zone (below the inferior pulmonary vein), and the middle lung zone (between the upper and the lower lung zone). Each of the six lung zones was scored according to the percentage of lung involvement as follows: score 0, no involvement; score 1, < 25% involvement; score 2, 25% to 50% involvement; score 3, 50% to 75% involvement; and score 4, >75% involvement. An overall lung score was calculated by summing the six lung zone scores, with values ranging from 0 to 24 for each patient.

### Statistical analysis

Data for categorical variables are expressed as frequency rates and percentages and were compared using the chi-square test. Continuous variables are described using the mean, median, and interquartile range (IQR), and differences between groups were compared using Student's t test (normally distributed data) or the Mann-Whitney U test (nonnormally distributed data). A receiver operating characteristic (ROC) curve was depicted to identify the optimal cutoff value for the worse outcome (need for mechanical ventilation or death). Univariate and multivariate logistic regression models using a back stepwise method were constructed to determine variables that were associated with an adverse outcome. First, variables were included to conduct the univariate analysis; Second, variables with *p*<0.10 were enrolled in the multivariate model with the backward stepwise method. Differences were considered significant at *p*<0.05 with a two-tailed test. The analyses were performed with statistical packages (SPSS 26.0; GraphPad Prism 8.2).

## Results

### Population characteristics

This study consisted of 224 patients with laboratory-confirmed COVID-19 who underwent chest CT examination at admission, including 120 (54%) males and 104 (46%) females, with a median age of 56 years (IQR: 41-70) (**Table 1**). Nearly half (44%) of the patients had comorbidities. Of 224 patients, 55 (25%) patients had hypertension, 33 (15%) patients had diabetes, 8 (4%) patients had chronic obstructive pulmonary disease (COPD) and 24 (11%) patients had coronary disease. Considering the clinical symptoms at admission, 200 (89%) patients had fever, 181 (81%) patients had cough, 80 (36%) patients had myalgia or fatigue, 45 (20%) patients had dyspnea, 35 (16%) patients had sore throat and 16 (7%) patients had diarrhea.

With regard to the laboratory test, the median white blood cell count and lymphocytes were 5.9×10^9^/L (IQR: 4.5-8.2) and 0.84×10^9^/L (IQR: 0.72-1.14), respectively, the median lactate dehydrogenase was 310 U/L (IQR: 239-430), and the median C-reactive protein (CRP) was 42.6 mg/L (IQR: 22.4-77.3).

Regarding clinical outcomes in our study, of 224 patients, 74 (33%) patients had adverse clinical outcomes [need for mechanical ventilation (74 patients) and death (18 patients)], while 150 (67%) patients had good outcomes.

### Initial CT manifestations

**Figure 1:**
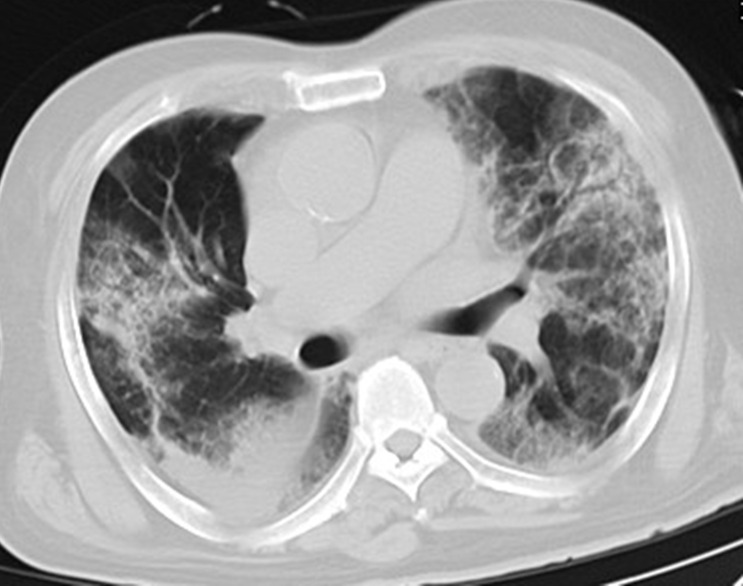
Initial chest CT of a 76-year-old man who presented with fever, cough, and dyspnea for 4 days. Bilateral extensive irregular patchy ground glass opacities (GGOs), strip opacities, consolidation, some with air bronchogram, with both central and peripheral distributions were observed. Mechanical ventilation treatments were used at admission, and the patient died of respiratory failure after 10 days of hospitalization.

**Figure 2:**
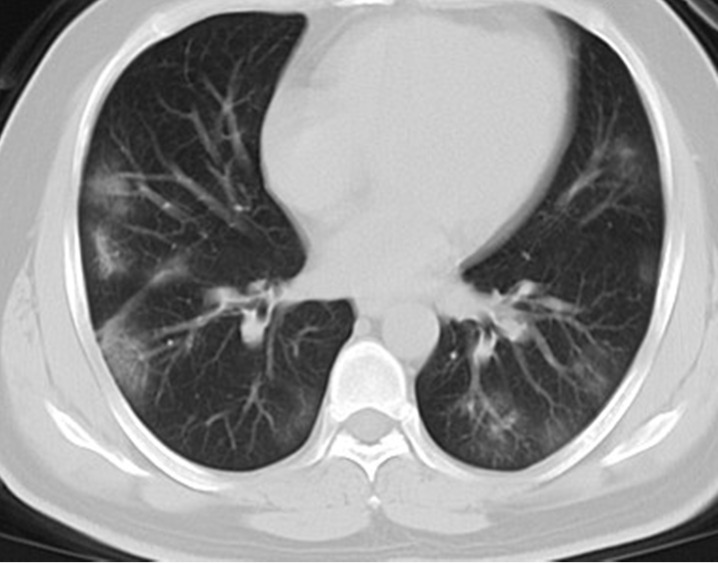
Initial chest CT of a 41-year-old man who presented with fever and cough for 3 days. Bilateral multiple patchy ground glass opacities (GGOs), with peripheral distribution were seen. The patient was discharged 6 days later after symptom improvement.

As shown in **Table 2**, all (100%) patients had abnormal CT findings at admission, 210 (94%) patients had bilateral lung involvement, and 102 (45%) patients had more than four lung zones involved. With regard to the distribution of lung lesions, 114 (51%) patients had peripheral distribution, 105 (47%) patients had both central and peripheral distribution, while only 5 (2%) patients had central distribution. Considering lung opacity, 210 (94%) patients had GGO, 46 (21%) patients had consolidation, 37 (17%) patients had GGO with consolidation, 137 (61%) patients had GGO with interstitial thickening, and 38 (17%) patients had air bronchogram (**Figure 1, Figure 2**). In addition, 2 (1%) patients had lymphadenopathy, and 3 (1%) patients had pleural effusion.

### Association with adverse clinical outcome

Clinical and laboratory variables correlated with an adverse outcome are presented in **Table 1.** There were significant differences in age, hypertension, diabetes, dyspnea, white blood cell counts, lymphocyte counts, lactate dehydrogenase levels and CRP levels between the groups. Age [67 (60-77) vs 50 (33-67) years, *p* < 0.001], the frequency of hypertension (42% vs 16%; *p* = 0.003), diabetes (28% vs 8%; *p* < 0.001), COPD (8% vs 1%; *p* = 0.010), dyspnea (32% vs 14%; *p* = 0.001), white blood cell counts (7.2×10^9^/L vs 5.1×10^9^/L; *p* < 0.001), lactate dehydrogenase levels (406 u/L vs 248 u/L; *p* < 0.001) and CRP levels (68.2 mg/L vs 28.4 mg/L; *p* < 0.001) were significantly higher and lymphocyte counts (0.66×10^9^/L vs 0.94×10^9^/L; *p* < 0.001) were lower in the patients who required mechanical ventilation or died.

CT variables associated with a poor outcome are presented in **Table 2.** Patients who were mechanically ventilated or died had a higher frequencies of more than four lung zones involved (73% vs 32%; *p* < 0.001), both central and peripheral distribution (57% vs 42%; p = 0.037), consolidation (27% vs 17%; *p* = 0.001), and air bronchogram (24% vs 13%; *p* = 0.039). Furthermore, the chest CT scores were higher (8.6±3.4 vs 5.4±2.1; *p* < 0.001) in the patients with worse clinical outcomes.

### Prognostic factors of adverse outcomes

**Figure 3:**
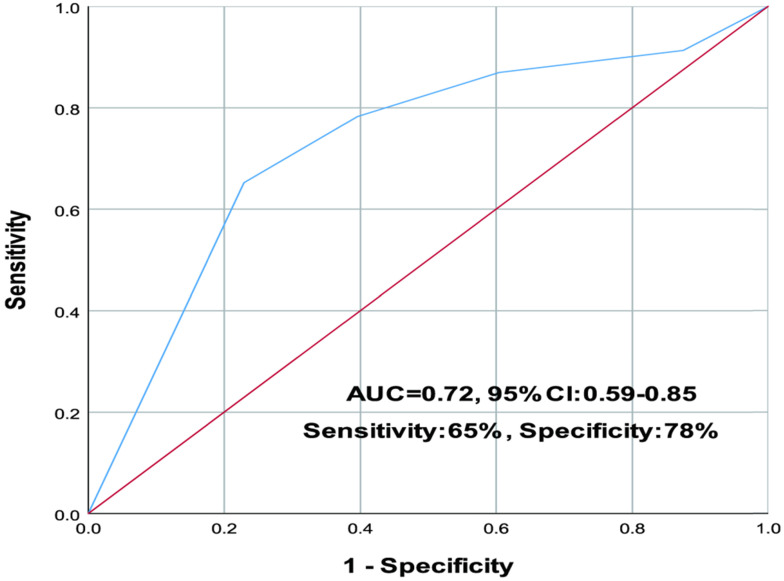
The involvement of more than four lung zones was identified as a cutoff with a sensitivity of 65% and a specificity of 78% for predicting poor prognosis. The area under the ROC (AUC) was 0.72 (95% confidence interval (CI): 0.59 to 0.85).

In the ROC analysis, the involvement of more than four lung zones was identified as a cutoff with a sensitivity of 65% and a specificity of 78% for predicting poor prognosis. The area under the ROC (AUC) was 0.72 (95% confidence interval (CI): 0.59 to 0.85) (**Figure 3**).

In the univariate logistic regression analysis, several parameters predicted worse clinical outcomes: age above 65 years [odds ratio (OR): 5.35; 95% CI: 1.62-17.47, *p* < 0.001], the presence of comorbidity (OR: 5.61; 95% CI: 0.67-12.23, *p* = 0.002), dyspnea (OR: 4.82; 95% CI: 1.14-15.31, *p* = 0.001), lymphocyte count (OR: 4.66; 95% CI: 1.12-14.40, *p* = 0.004), CRP level (OR: 4.91; 95% CI: 1.05-16.87, *p* = 0.010), lactate dehydrogenase level (OR: 2.01; 95% CI: 0.68-6.47, *p* < 0.001), and the involvement of more than four lung zones (OR: 4.21; 95% CI: 1.24-18.19, *p* = 0.001) (**Table 3**).

Multivariate analysis demonstrated that age above 65 years (OR: 3.65; 95% CI: 1.11-10.59, *p* < 0.001), the presence of comorbidity (OR: 5.20; 95% CI: 1.64-19.22, *p* = 0.001), dyspnea (OR: 3.19; 95% CI: 1.35-8.46, *p* = 0.013), and the involvement of more than four lung zones (OR: 3.93; 95% CI: 1.44-12.89, *p* = 0.006) were independently associated with a worse outcome.

## Discussion

The present study explored the prognostic factors of patients with COVID-19 associated with poor outcomes. We found that clinical and initial CT variables correlated with adverse outcomes. Older age, the presence of comorbidity, dyspnea, and the involvement of more than four lung zones on initial chest CT were associated with the need for mechanical ventilation or death. To the best of our knowledge, this is the first study to report the involvement of more than four lung zones on initial chest CT were associated with the need for mechanical ventilation or death.

We found that the clinical variables of older age and the presence of comorbidities were independently associated with a worse outcome in patients with COVID-19, and our results were consistent with earlier studies 13-15. In fact, it is not surprising that older age and pre-existing illness increases the risk of death or a complicated course for many diseases. Furthermore, the present study demonstrated that dyspnea is predictive of poor outcome in patients with COVID-19. The result is also in line with a previous report in patients with COVID-19 16 and in influenza A (H1N1) 17. Therefore, paying more attention to older patients with COVID-19 with preexisting conditions and dyspnea at admission in clinical practice may be important for improving the outcome.

In our study, the extent of lung involvement on chest CT is associated with disease severity and clinical outcomes in patients with COVID-19. CT has been demonstrated to be helpful for early diagnosis and monitoring in patients with COVID-19 pneumonia. Similar to previous studies 6,7, bilateral involvement, GGO, consolidation, and GGO with interstitial thickening were the most common initial CT findings of COVID-19 in our study. Bilateral extensive opacities on initial chest radiographs are a prognostic factor of adverse outcomes in patients with SARS 9, influenza A (H1N1) 10, and community-acquired pneumonia 18,19. Similarly, we found that patients' with COVID-19 with the involvement of more than four lung zones is a prognostic factor of adverse outcomes. Moreover, we found higher white blood cell counts, lactate dehydrogenase levels and CRP levels and more marked lymphopenia in patients with the involvement of more lung zones, which may suggest severe SARS-CoV-2 infection.

Clinically, Two types [low (L) and high (H) elastance phenotypes] can be distinguished in mechanically ventilated patients, based on lung mechanics, ventilation-to-perfusion ratio, and CT scans 20. The L-type is characterized by the normal lung weight (only ground-glass densities on CT), low elastance (highly compliant lungs with nearly normal gas volumes), low V/Q-ratio due to loss of perfusion regulation and hypoxic vasoconstriction, and low lung recruitability because of the low amount of nonaerated lung tissue. The H-type, with increased pulmonary edema (consolidations and GGOs), leads to high lung weight and decreased lung compliance (high elastance) 21. In our study, the patients who had ventilation tended to have mixed pattern (consolidations and GGOs) and to have more than four lung zones involvement, suggesting the H-type had adverse outcomes.

COVID-19 is a highly contagious disease caused by SARS-CoV-2, which is correlated with highly morbidity and mortality. Our study reveals that the clinical evidence of older age, the presence of comorbidity, dyspnea, and initial chest CT evidence of extensive lung zones involvement may have significance for predicting clinical outcomes in patients with COVID-19. Patients with the abovementioned evidence had higher risk of worse outcomes. It may be valuable for clinicians to evaluate the possibility of adverse outcomes at admission for the management of this acute infectious disease.

Several limitations in the present study should be noted. First, this is a single-center retrospective study, making it difficult to avoid selection bias. Second, most patients with adverse outcomes were elderly individuals in our study. Thus, a study population including younger severe patients' would be important to provide a better generalization of the results in a future study.

## Conclusion

Initial chest CT may be helpful for predicting the clinical outcomes in patients with COVID-19. Patients with the involvement of more lung zones and higher CT scores on initial CT were significantly associated with a higher incidence of adverse outcomes. Older age, the presence of comorbidity, dyspnea and the involvement of more than four lung zones on initial chest CT were independent predictors of a worse outcome.

## Figures and Tables

**Table 1:** Clinical characteristics of the patients with COVID-19

Variable	Total (n=224)	Without Adverse Outcome (n=150)	With Adverse Outcome (n=74)	*P* value
Age (years)	56 (41-70)	50 (33-67)	67 (60-77)	<0.001
Men	120 (54)	76 (51)	44 (59)	0.215
**Comorbidities**				
Hypertension	55 (25)	24 (16)	31 (42)	<0.001
Diabetes	33 (15)	12 (8)	21 (28)	<0.001
COPD	8 (4)	2 (1)	6 (8)	0.010
Coronary disease	24 (11)	10 (7)	14 (19)	0.005
**Initial symptoms**				
Fever	200 (89)	132 (88)	68 (92)	0.376
Cough	181 (81)	122 (81)	59 (82)	0.774
Myalgia or fatigue	80 (36)	46 (31)	34 (46)	0.025
Dyspnea	45 (20)	21 (14)	24 (32)	0.001
Sore throat	35 (16)	25 (17)	10 (14)	0.780
Diarrhea	16 (7)	10 (7)	6 (8)	0.694
**Laboratory data**				
White blood cell count (×10^9^/L)	5.9 (4.5-8.2)	5.1 (4.2-6.8)	7.2 (5.1-9.6)	0.001
Lymphocyte count (×10^9^/L)	0.84 (0.72-1.14)	0.94 (0.62-1.33)	0.66 (0.56-0.88)	<0.001
Lactate dehydrogenase (U/L)	310 (239-430)	248 (198-310)	406 (317-598)	<0.001
C-reactive protein (mg/L)	42.6 (22.4-77.3)	28.4 (10.4-52.8)	68.2 (41.2-109.6)	<0.001
Mechanical Ventilation	74 (33 )	0 (0)	74 (100 )	<0.001
Duration of ventilation, days	-	0 (0)	6 (3-9)	<0.001
Death	18 (8)	0 (0)	18 (24 )	<0.001

Data are expressed as the median (interquartile range) or n (%). COPD, chronic obstructive pulmonary disease.

**Table 2:** Comparison of clinical outcomes of patients with COVID-19 with initial abnormal chest CT

Characteristic	Total (n=224)	Without Adverse Outcome (n=150)	With Adverse Outcome (n=74)	*P* value
Bilateral involvement	210 (94)	138 (92)	72 (97)	0.123
More than four lung zones involved	102 (45)	48 (32)	54 (73)	<0.001
**Distribution**				
Central	5 (2)	3 (2)	2 (3)	0.638
Peripheral	114 (51)	84 (56)	30 (40)	0.029
Both central and peripheral	105 (47)	63 (42)	42 (57)	0.037
**Opacity**				
GGO	210 (94)	142 (95)	68 (92)	0.420
Consolidation	46 (21)	26 (17)	20 (27)	0.001
GGO with consolidation	37 (17)	23 (15)	14 (19)	0.091
GGO with interstitial thickening	137 (61)	88 (59)	49 (66)	0.276
Air bronchogram	38 (17)	20 (13)	18 (24)	0.039
**Other findings**				
Lymphadenopathy	2 (1)	1 (1)	1 (1)	0.608
Pleural effusion	3 (1)	1 (1)	2 (3)	0.212
Chest CT score	6.2±2.6	5.4±2.1	8.6±3.4	<0.001

Data are expressed as the mean ± standard deviation or n (%). CT, computed tomography; GGO, ground glass opacity.

**Table 3:** Univariate analysis for prognostic factors of adverse outcome in patients with COVID-19 using logistic regression analysis

Variable	OR (95% CI)	*P* value
Age > 65 years	5.35 (1.62-17.47)	< 0.001
Presence of comorbidity	5.61 (0.67-12.23)	0.002
Dyspnea	4.82 (1.14-15.31)	0.001
White blood cell count	1.49 (1.05-2.01)	0.149
Neutrophil count	1.71 (1.21-2.37)	0.092
Lymphocyte count	4.66 (1.12-14.40)	0.004
C-reactive protein	4.91 (1.05-16.87)	0.010
Lactate dehydrogenase	2.01 (0.68-6.47)	< 0.001
More than four lung zones involved	4.21 (1.24-18.19)	0.001

OR, odds ratios; CI, confidence intervals.

**Table 4:** Multivariate analysis for prognostic factors of adverse outcome in patients with COVID-19 using logistic regression analysis

Variable	OR (95% CI)	*P* value
Age > 65 years	3.65 (1.11-10.59)	< 0.001
Presence of comorbidity	5.20 (1.64-19.22)	0.001
Dyspnea	3.19 (1.35-8.46)	0.013
More than four lung zones involved	3.93 (1.44-12.89)	0.006

See Table 3 for definitions of abbreviations.

## Abbreviations

COVID-19: coronavirus disease 2019
COPD: chronic obstructive pulmonary disease
CRP: C-reactive protein
CT: computed tomography
GGO: ground glass opacity
SARS-CoV-2: severe acute respiratory syndrome coronavirus 2
